# Analysis of the Association between the Tgfb1 Gene Haplotype and Liver Diseases in Children

**DOI:** 10.32607/actanaturae.19425

**Published:** 2023

**Authors:** R. M. Kurabekova, O. E. Gichkun, O. M. Tsirulnikova, I. E. Pashkova, V. A. Fomina, O. P. Shevchenko, S. V. Gautier

**Affiliations:** V.I. Shumakov National Medical Research Center of Transplantology and Artificial Organs, Moscow, 123182 Russian Federation; I.M. Sechenov First Moscow State Medical University (Sechenov University), Moscow, 119991 Russian Federation

**Keywords:** congenital and inherited liver diseases, biliary atresia and hypoplasia, pediatric liver recipients, liver transplantation

## Abstract

Transforming growth factor-β1 (TGF-β1), a cytokine with immunosuppressive and pro-fibrogenic activity, is a potential marker of infection, liver transplant rejection, and fibrosis. Its levels in the blood and tissues depend on many factors; however, the role of gene polymorphism is still unclear. In this work, the distribution frequency of three single nucleotide polymorphism (SNP) variants of the *Tgfb1 *gene, namely rs1800469, rs1800470, and rs1800471, was studied in children with end-stage liver disease (ESLD). The study included 225 pediatric liver recipients aged 1 month to 16 years (median, 8 months), including 100 boys and 125 girls, and 198 healthy individuals aged 32.7 ± 9.6 years, including 78 men and 120 women. The indication for liver transplantation in children was ESLD, which was mostly caused by congenital and inherited liver diseases. SNPs were detected by real-time polymerase chain reaction using TaqMan probes and DNA isolated from peripheral blood. SNP frequency distribution was in Hardy–Weinberg equilibrium and did not differ between children with liver diseases and the healthy ones. Analysis of the SNPs frequency based on allelic interaction models did not reveal any differences between patients and the healthy individuals. Evaluation of linkage disequilibrium for *Tgfb1 *polymorphic variant pairs revealed a statistically significant linkage between all studied variants. Seven haplotypes, which are variants of SNP combinations, were observed in the studied groups of patients and healthy individuals. A total of 80% of the group had three haplotypes, whose frequencies did not differ between patients and the healthy individuals. Significant differences were found in the frequency of the haplotypes A-A-C, G-G-C, and G-A-G (at rs1800469, rs1800470, and rs1800471, respectively), which were observed up to 11 times more often in recipients compared to the healthy individuals. It is possible that these haplotypes are ESLD-predisposing variants, which may also contribute to the development of complications after liver transplantation in children.

## INTRODUCTION

Liver transplantation is the only effective treatment for children with end-stage liver disease (ESLD) [1]. Despite advances in organ transplantation and the high survival rates of recipients, the period after transplantation can be accompanied by such complications as infections, transplant rejection, and fibrosis. Prevention of complications requires the use of accurate approaches to their prediction and diagnosis using molecular and genetic markers.

Transforming growth factor-β1 (TGF-β1), an important component of the immune system with immunosuppressive and pro-fibrogenic activities, plays an essential role in the development of complications after organ transplantation [2, 3].

A series of studies, including our work, showed that the TGF-β1 cytokine level in pediatric liver recipients correlates with the graft function and has a prognostic and diagnostic significance [4, 5, 6]. TGF-β1 levels in recipient’s blood and tissues are determined by numerous factors, including genetic ones. Considering that ESLDs in children are mainly congenital and hereditary, this genetic factor can play some role in both the progression of the disease and emergence of posttransplant complications.

*Tgfb1 *contains a series of single nucleotide polymorphisms (SNPs), which can be associated with various pathologies [7, 8, 9]. The greatest interest of researchers specializing in the field of solid organ transplantation has been drawn to the following three SNPs in *Tgfb1*: rs1800469 with a C > T substitution in the promoter region (C(–509)T); rs1800470 with a T > C substitution in codon 10 of the first exon (T(+869)C), resulting in Leu-to-Pro substitution in the protein; and rs1800471 with a C > G substitution in codon 25 of the first exon (C(+915)G), leading to a Arg-to-Pro substitution in the protein. The rs1800469 polymorphism is located in the promoter region and affects the recruitment of transcription factors, thus disrupting transcription regulation. SNPs rs1800470 and rs1800471 are located in the first exon and affect protein expression. These SNPs are considered to be the cause of the differences in the TGF-β1 activity level in tissues and can be associated with the development of post-transplant complications [10, 11, 12]. The role of the *Tgfb1 *polymorphism in pediatric liver diseases is still unknown.

The aim of this study is to evaluate the frequencies of three *Tgfb1 *SNPs in young children with ESLD.

Establishment of the role of the polymorphism of the genes determining the activity of pro- and anti- inflammatory cytokines, including TGF-β1, in the pathogenesis of various diseases in solid organ recipients will make it possible to both predict the risk of developing the disease and its severity and select a therapeutic approach for an individual patient. An example of polymorphism analysis in clinical practice is the genotyping of human major histocompatibility complex genes and further selection of a recipientcompatible donor organ for transplantation. Another example is the polymorphism of *CYP3A5*, which encodes a member of the cytochrome P450 superfamily that can disrupt functional protein synthesis and exert a significant effect on the clearance of the immunosuppressive drug tacrolimus. Selection of a tacrolimus daily dose that takes into account the *CYP3A5* genotype allows one to attain the desired drug concentration.

## EXPERIMENTAL

The study protocol was approved by the local ethics committee of the V.I. Shumakov National Medical Research Center of Transplantology and Artificial Organs. Either patients or their guardians signed a written informed consent to participate in the study. The consent is stored in the patient’s medical records.

The study included 225 pediatric liver recipients (100 boys and 125 girls) aged 1–192 months (16 years; median, 8 months) and 198 healthy individuals aged 32.7 ± 9.6 years (78 males and 120 females). This sample was considered an open Russian population, since the ethnicity of the study participants was not identified.

Liver diseases in patients included the following pathologies: biliary atresia (BA), Caroli disease, biliary hypoplasia (BH), Alagille syndrome, Byler disease, and other rare liver diseases like Crigler–Najjar syndrome, von Gierke disease, alpha-1 antitrypsin deficiency, tyrosinemia, fulminant autoimmune hepatitis, cryptogenic cirrhosis, etc. The demographic and clinical characteristics of the pediatric liver recipients included in the study are presented in *Table 1*.

The patients included in the study underwent transplantation of a liver fragment from a living related donor. Recipients received two- and three-component immunosuppressive therapy, which included tacrolimus, corticosteroids, and mycophenolate drugs. Routine examination and treatment of patients were conducted in accordance with the clinical recommendations of the Russian Transplant Society and the protocols of the V.I. Shumakov National Medical Research Center of Transplantology and Artificial Organs.

**Table 1:** Patients included in the study

Characteristic	Value
Number of patients, n	225
Age, months	8 (1–192)
Male/female ratio (%)	100(44)/125(56)
Disease, number of cases (%)	
BA BH Caroli disease Alagille syndrome Byler disease Others	107(48) 24(11) 11(5) 12(5) 10(4) 61(27)

BA – biliary atresia;

BH – biliary hypoplasia.

Genomic DNA was isolated from peripheral blood using a QIAamp DNA Blood Mini Kit (Qiagen, Germany) on an automated QIAcube™ system (Qiagen, Germany) according to the manufacturer’s instructions. The *Tgfb1 *polymorphic variants rs1800469, rs1800470, and rs1800471 were analyzed by real-time polymerase chain reaction using TaqMan probes (Applied Biosystems, USA) on a CFX96™ realtime PCR detection system (Bio-Rad, USA) according to the manufacturer’s instructions.

The statistical analysis was performed using the Microsoft Excel software. The distribution of the studied SNP genotype frequencies, haplotype structure, and pairwise linkage disequilibrium were analyzed using the SNPstats software [13]. In order to confirm the independent distribution of the studied polymorphic alleles, we tested them for compliance with the Hardy–Weinberg law. Allele frequency was calculated using the following formula: allele frequency = ((2 × number of homozygotes) + number of heterozygotes))/2 × total number of individuals. The frequencies of the genotypes and individual alleles were compared between different groups using the Pearson χ2 criterion. To quantitatively represent the impact of a possible genotype on a certain characteristic, odds ratios (ORs) and their 95% confidence intervals (CI) were calculated. To assess the linkage disequilibrium, *D*-statistics and the correlation coefficient *r* were calculated. The critical significance level was considered 0.05.

## RESULTS

**Fig. 1:**
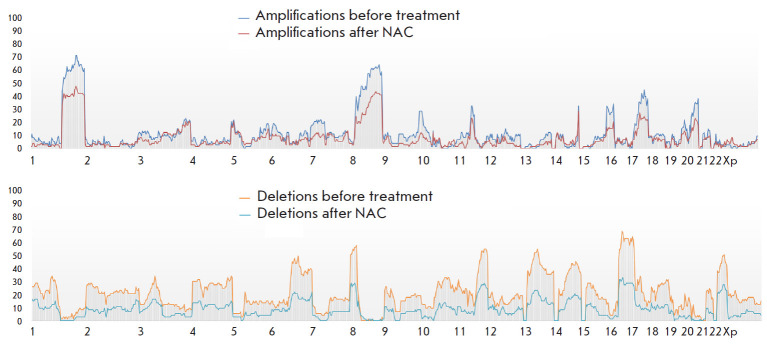
Frequency distributions of *Tgfb1 *SNPs rs1800469 (*A*), rs1800470 (*B*), and rs1800471 (*C*) in children with liver diseases and healthy individuals

Three *Tgfb1 *polymorphic variants (rs1800469, rs1800470, and rs1800471) were genotyped in the DNA of the studied patients. The frequencies of the various genotypes and alleles were calculated. *Figure 1* presents the distribution of the genotypes and alleles in children with liver diseases and healthy individuals.

A comparative analysis of the frequencies of the studied genotypes and alleles in the children with liver diseases and healthy donors did not reveal any statistically significant differences.

No gender-related statistically significant differences in the distribution of the studied SNPs were found between the patients and healthy individuals. At the same time, significant differences were observed in the frequency of the rare genotype C/G rs1800471 between young female patients and healthy women; this genotype was 3.96 times more common in girls than in healthy women (OR, 3.96, 95% CI 1.09–14.43,* p *= 0.01).

The frequency distribution of the studied genotypes of all three polymorphic variants was in agreement with the Hardy–Weinberg equilibrium in both the patients and healthy individuals (*Table 2*).

**Table 2:** Compliance of Tgfb1 polymorphism distributions with the Hardy–Weinberg law

Group	rs1800469		rs1800470		rs1800471	
	χ^2^	p	χ^2^	p	χ^2^	p
Healthy individuals	0.4779	0.54	0.0166	0.898	0.0842	0.772
Patients	0.0013	0.971	2.6579	0.120	0.3925	0.531

Note: p < 0.05 do not meet the Hardy–Weinberg equilibrium.

A comparative analysis of the distribution of the genotype and allele frequencies of three *Tgfb1 *SNPs using different models of allelic interactions (codominant, dominant, recessive, and superdominant) was conducted in healthy individuals and pediatric liver recipients. For each model, OR and the error rate were calculated by comparing children with liver diseases and healthy individuals (*Table 3*).

**Table 3:** Comparison of Tgfb1 polymorphism distributions in children with liver diseases and healthy individuals using different inheritance models

Model	Genotype	Frequency, recipients	Frequency, healthy individuals	OR (95% CI)	p
rs1800469					
Codominant	G/G	85 (38%)	74 (39.8%)	1.00	0.71
	A/G	106 (47.3%)	90 (48.4%)	1.02 (0.67–1.55)	
	A/A	33 (14.7%)	22 (11.8%)	1.29 (0.69–2.41)	
Dominant	G/G	85 (38%)	74 (39.8%)	1.00	0.73
	A/G-A/A	139 (62%)	112 (60.2%)	1.07 (0.72–1.60)	
Recessive	G/G-A/G	191 (85.3%)	164 (88.2%)	1.00	0.41
	A/A	33 (14.7%)	22 (11.8%)	1.27 (0.71–2.28)	
Superdominant	G/G-A/A	118 (52.7%)	96 (51.6%)	1.00	0.83
	A/G	106 (47.3%)	90 (48.4%)	0.96 (0.65–1.41)	
rs1800470					
Codominant	A/A	91 (40.8%)	74 (39.8%)	1.00	0.49
	A/G	94 (42.1%)	87 (46.8%)	0.87 (0.57–1.33)	
	G/G	38 (17%)	25 (13.4%)	1.23 (0.68–2.22)	
Dominant	A/A	91 (40.8%)	74 (39.8%)	1.00	0.81
	A/G-G/G	132 (59.2%)	112 (60.2%)	0.95 (0.64–1.42)	
Recessive	A/A-A/G	185 (83%)	161 (86.6%)	1.00	0.32
	G/G	38 (17%)	25 (13.4%)	1.32 (0.76–2.28)	
Superdominant	A/A-G/G	129 (57.9%)	99 (53.2%)	1.00	0.33
	A/G	94 (42.1%)	87 (46.8%)	0.82 (0.56–1.22)	
rs1800471					
Codominant	C/C	205	(91.9%) 180	(96.3%) 1.00	0.063
	C/G 18	(8.1%)	7 (3.7%)	2.26 (0.92–5.53)	

No statistically significant differences were found in the distribution of the studied *Tgfb1 *variant frequencies between patients and healthy individuals using different allelic interaction models. In addition, no significant gender-related differences in genotype distribution were noted.

Since the analyzed loci are located on the same chromosome, linkage disequilibrium (LD), i.e., linked locus inheritance and haplotype formation, can be observed. *Table 4* presents the results of the statistical analysis of pairwise linkage of the studied *Tgfb1 *variants as *D*, *D*’, and *r*-statistics, including the error rate.

A statistically significant linkage was found between all studied variants. The strongest linkage was observed for the first pair of loci (rs1800469– rs1800470); the weakest linkage was found between the other two pairs.

**Table 4:** Statistical assessment of the linkage disequilibrium for pairs of polymorphic variants of the Tgfb1 gene

SNP pair	D	D’	r	p
rs1800469–rs1800470	0.1447	0.6259	0.6184	0
rs1800469–rs1800471	-0.0113	0.9934	-0.136	0.0001
rs1800470–rs1800471	0.0089	0.4628	0.1062	0.0021

Seven combinations of the studied SNPs were found in the studied groups. *Table 5* presents the identified haplotypes in order of decreasing frequency, frequencies of various groups, the OR between healthy individuals and recipients, and the OR error rate.

**Table 5:** Tgfb1 haplotype frequencies in children with liver diseases and healthy individuals

No.	Nucleotide-			Frequency			Odds ratio (95% CI)	p
	rs1800469	rs1800470	rs1800471	total	patients	healthy individuals		
1	G	A	C	0.5236	0.4680	0.5864	1.00	
2	A	G	C	0.2841	0.2410	0.3190	1.05 (0.75–1.47)	0.76
3	A	A	C	0.0862	0.1340	0.0374	3.12 (1.72–5.67)	0.0002*
4	G	G	C	0.0754	0.1170	0.0371	2.88 (1.56–5.32)	0.0008*
5	G	G	G	0.0180	0.0154	0.0176	1.54 (0.51–4.65)	0.44
6	G	A	G	0.0127	0.0174	0.0026	11.18 (1.37–91.18)	0.025*
7	A	G	G	0.0038	0.0073	0	–	–

^*^p < 0.05.

*Table 5* shows that the combination of G-A-C alleles is the most prevalent (about 50% of cases in patients and 60% of cases in healthy individuals), whose distribution does not differ significantly between the groups. The second most common haplotype, A-G-C, is present in approximately 30% of the studied groups; its frequency also did not differ between the patients and healthy individuals. The frequencies of the fifth most common haplotype, G-G-G, also did not differ between the groups: there were about 2% of cases in both groups. In general, about 80% of the studied individuals had three out of seven haplotype variants with the same frequencies in recipients and healthy individuals.

Statistically significant differences in the frequency of the least common haplotypes were found. These haplotypes are more prevalent in recipients compared to healthy individuals. Haplotypes No. 3 (A-A-C), No. 4 (G-G-C), and No. 6 (G-A-G) are 3.12 (*p*= 0.0002), 2.88 (*p *= 0.0008), and 11.18 (*p *= 0.025) times more prevalent in patients than in healthy individuals, respectively. In general, the less common haplotypes No. 3, 4, and 6 were found in 26.84% of the patients and 7.71% of the healthy individuals. The least common haplotype A-G-G (No. 7) was practically absent in healthy individuals, while its frequency in recipients was < 1%, which made it impossible to compare the groups for this parameter.

## DISCUSSION

The development of approaches to the non-invasive diagnosis of post-transplant complications is important in relation to pediatric liver recipients due to the high risk of invasive procedures. Gene diagnosis has such important advantages over other methods as independence from the physiological state, immutability, and the possibility to perform only a single test. The results of genetic tests can provide information on a patient’s predispositions and allow for the use of personalized therapy by selecting drugs based on an individual patient’s characteristics.

In this work, we analyzed the frequencies of the three most prevalent *Tgfb1 *SNPs in children with ESLD and healthy individuals in an open Russian population. We showed that the distribution of these SNPs does not differ between patients and healthy individuals and meets the Hardy–Weinberg equilibrium.

The data we obtained did not reveal an association between different *Tgfb1 *SNPs and pediatric liver diseases. We have not found publications on the study of the *Tgfb1 *genetic polymorphism in young children with congenital and hereditary liver diseases in Russian and other populations. The role of* Tgfb1 *SNPs in the development of various liver pathologies has been studied in adult patients; however, the results of these studies are inconclusive [7 , 8, 9, 10, 11, 12]. On the one hand, there is data indicating an association between these SNPs and transplant rejection and chronic dysfunction [10, 11, 12]. On the other hand, there are also studies that did not uncover any association between the *Tgfb1 *polymorphism and both transplant rejection and donor liver fibrosis in adult patients [14, 15, 16].

The frequencies of SNPs rs1800469, rs1800470, and rs1800471 in healthy individuals identified in our study are consistent with the data of other domestic authors [17, 18]. A comparison of the distribution of the allele frequencies studied in our work with the data deposited into the U.S. National Center for Biotechnology Information (NCBI) also did not reveal significant differences from that of *Tgfb1 *in the European population: rs1800469 – A(37%)/G(63%); rs1800470 – A(56%)/G(44%); and rs1800471 – C(94%)/G(6%).

The TGF-β1 cytokine is a vital protein involved in the regulation of the key cellular processes; therefore, a significant impairment of its function can be fatal [19]. It is possible that individual single nucleotide substitutions have a limited effect on the protein function, while a combination of several substitutions can have a pronounced effect. Therefore, analysis of the haplotypes of several SNPs can be more informative than a study of a single nucleotide substitution.

We noticed a linkage disequilibrium of SNPs rs1800469, rs1800470, and rs1800471, which form seven haplotype variants, in patients and healthy individuals. The prevalence of the three most common haplotypes did not vary significantly between patients and healthy individuals. The analysis of the same *Tgfb1 *haplotypes conducted by other authors showed a similar prevalence of the most common haplotype, G-A-C, in healthy individuals, which was about 50–60% [17, 20].

The least common haplotypes identified in our study were more prevalent in ESLD patients compared to the healthy individuals. This suggests involvement of these haplotypes in the predisposition to liver diseases. A significant number of diseases in the studied patients are congenital and hereditary, while the genetic nature of the majority has not been studied in detail. Therefore, a search for disease-associated haplotypes can be of scientific and practical value in transplantation. It is possible that the identified haplotypes not only predispose children to the liver disease, but also contribute to the complications that emerge after liver transplantation. However, further studies are required to unambiguously establish such a causal relationship.

The study design is based on the case–control method, which imposes certain limitations on the legitimacy of establishing a causal relationship between the identified associations. It should be noted that it is not always possible to unambiguously determine the haplotype based on the genotype using PCR. Only sequencing allows for accurate haplotype identification.

The limitation in the conclusion on a possible association between the studied haplotypes and predisposition to ESLD is also due to the fact that some pathologies can be determined by numerous genetic factors/polymorphisms, each of which makes only a small contribution to the overall risk of developing the disease, while their significance is difficult to evaluate when analyzing a small patient sample. For instance, the functional activity of TGF-β1 can be determined by not only the gene polymorphism, but also by the genetic variants of the other factors participating in the cytokine pathway, such as binding proteins and receptors. The risk of developing hepatitis C after transplantation in patients was shown to be associated with the frequency of the SNP rs868 located in the non-coding 3′-UTR region of the TGF-β1 receptor gene (*Tgfbr1*) [21]. In addition, the interaction of different genes can have a clinical significance. An association between *HLA *genes and the genes of various cytokines, including TGF-β1, was found in patients with such a multifactorial autoimmune disease as type 1 diabetes mellitus, in which polymorphisms of the human major histocompatibility complex genes may play an important role [22]. Some combinations of polymorphic variants of the cytokine TNF-α, IFN-γ, IL-6, and TGF-β1 genes were shown to be less common in patients with type 1 diabetes mellitus compared to the control, which suggested a protective role for these haplotypes [22]. Linkage disequilibrium of the TNF-α variant characteristic of the protective haplotype with two polymorphic HLA variants was also noted.

Predisposition to various polygenic diseases can also be determined by an individual’s ethnicity, which points to the need to study ethnically homogeneous groups. However, we did not determine the ethnicity of the individuals in our study. Therefore, the obtained results can be considered valid for an open Russian population.

## CONCLUSIONS

The level of the multifunctional cytokine TGF-β1 is a potential biomarker of infection, transplant rejection, and fibrosis. In this work, we studied the distribution of the three most significant *Tgfb1* polymorphisms (rs1800469, rs1800470, and rs1800471) in pediatric patients with congenital and hereditary liver diseases. We demonstrated that the frequency of each individual polymorphism does not differ significantly from that of healthy individuals and meets the Hardy– Weinberg equilibrium.

However, the frequency of haplotypes of the three studied *Tgfb1 *polymorphisms differs statistically significantly between patients and healthy individuals. Seven different haplotypes were found in the studied group. Of them, three were observed 3 to 11 times more often in recipient children compared to healthy ones. These haplotypes, namely A-A-C, G-G-C, and G-A-G, which correspond to rs1800469, rs1800470, and rs1800471, respectively, can be associated with predisposition to end-stage liver disease in children. Additional studies are warranted in order to further elucidate the role of these haplotypes in post-transplant complications.

## Abbreviations

TGF-β1: transforming growth factor β1
Tgfb1: TGF-β1 gene
SNP: single nucleotide polymorphism
ESLD: end-stage liver disease
OR: odds ratio
BA: biliary atresia
BH: biliary hypoplasia
LD: linkage disequilibrium
HLA: human leukocyte antigen
